# A randomised controlled trial comparing standard or intensive management of reduced fetal movements after 36 weeks gestation-a feasibility study

**DOI:** 10.1186/1471-2393-13-95

**Published:** 2013-04-16

**Authors:** Alexander EP Heazell, Giovanna Bernatavicius, Stephen A Roberts, Ainslie Garrod, Melissa K Whitworth, Edward D Johnstone, Joanna C Gillham, Tina Lavender

**Affiliations:** 1 Maternal and Fetal Health Research Centre, Manchester Academic Health Science Centre, University of Manchester, Manchester, UK; 2 Centre for Biostatistics, Institute of Population Health, Manchester Academic Health Science Centre, University of Manchester, Manchester, UK; 3 St Mary’s Hospital, Oxford Road, Manchester, UK; 4 School of Nursing, Midwifery and Social Work, University of Manchester, Manchester, UK; 5 Maternal and Fetal Health Research Centre, 5th floor (Research), St Mary’s Hospital, Oxford Road, Manchester M13 9WL, UK

**Keywords:** Reduced fetal movements, Randomised controlled trial, Human placental lactogen, Feasibility, Maternal anxiety

## Abstract

**Background:**

Women presenting with reduced fetal movements (RFM) in the third trimester are at increased risk of stillbirth or fetal growth restriction. These outcomes after RFM are related to smaller fetal size on ultrasound scan, oligohydramnios and lower human placental lactogen (hPL) in maternal serum. We performed this study to address whether a randomised controlled trial (RCT) of the management of RFM was feasible with regard to: i) maternal recruitment and retention ii) patient acceptability, iii) adherence to protocol. Additionally, we aimed to confirm the prevalence of poor perinatal outcomes defined as: stillbirth, birthweight <10^th^ centile, umbilical arterial pH <7.1 or unexpected admission to the neonatal intensive care unit.

**Methods:**

Women with RFM ≥36 weeks gestation were invited to participate in a RCT comparing standard management (ultrasound scan if indicated, induction of labour (IOL) based on consultant decision) with intensive management (ultrasound scan, maternal serum hPL, IOL if either result was abnormal). Anxiety was assessed by state-trait anxiety index (STAI) before and after investigations for RFM. Rates of protocol compliance and IOL for RFM were calculated. Participant views were assessed by questionnaires.

**Results:**

137 women were approached, 120 (88%) participated, 60 in each group, 2 women in the standard group did not complete the study. 20% of participants had a poor perinatal outcome. All women in the intensive group had ultrasound assessment of fetal size and liquor volume vs. 97% in the standard group. 50% of the intensive group had IOL for abnormal scan or low hPL after RFM vs. 26% of controls (p < 0.01). STAI reduced for all women after investigations, but this reduction was greater in the standard group (p = 0.02). Participants had positive views about their involvement in the study.

**Conclusion:**

An RCT of management of RFM is feasible with a low rate of attrition. Investigations decrease maternal anxiety. Participants in the intensive group were more likely to have IOL for RFM. Further work is required to determine the likely level of intervention in the standard care arm in multiple centres, to develop additional placental biomarkers and to confirm that the composite outcome is valid.

**Trial registration:**

ISRCTN07944306

## Background

Reduced fetal movements (RFM) is a frequently seen problem in maternity care with 6–15% of women reporting attending at least one occasion of RFM to health professionals in the third trimester of pregnancy [1,2]. RFM, defined by maternal perception of significantly reduced or absent fetal activity, is associated with increased risk of stillbirth and fetal growth restriction (FGR) due to placental dysfunction [3,4]. Despite this association there is a paucity of evidence to direct clinical management of women presenting with RFM. This has been recently highlighted by guidelines from the Royal College of Obstetrics and Gynaecology (RCOG) and a meta-analysis, where the highest level of evidence identified was level 2 (case-control study) [5-7]. The absence of high-quality evidence has led to wide variation in management strategies for RFM in high-income settings [8,9].

Cohort studies carried out on different populations have found that the investigations that best predicted poor perinatal outcome (including stillbirth, FGR and admission to the neonatal intensive care unit (NICU)) after RFM were electronic fetal monitoring, ultrasound assessment of fetal weight and liquor volume and measurement of human placental lactogen (hPL) in maternal serum [6,10]. Although there are randomised controlled trials (RCT) of counting fetal movements by a formal structure (e.g. count to ten [11,12]) there have been no published RCTs of patient management following presentation with RFM [7]. To undertake an RCT of patient management raises important practical concerns including: maternal anxiety for fetal wellbeing, the need to make a decision regarding participation in a short period of time due to the acute nature of RFM and adherence to protocol. Thus, studies have adopted an approach of changing practice at the unit level in quality-improvement projects [6] or stepwise cluster RCT (AFFIRM, NCT01777022 [13]).

We performed this study to address whether an RCT of the management of RFM in individual patients was an appropriate trial design, and was feasible with regard to i) maternal recruitment and retention ii) patient acceptability, iii) adherence to protocol. In addition, we wished to confirm the prevalence of poor perinatal outcomes in the study population.

## Methods

Women attending a single maternity unit (St Mary’s Hospital, Manchester, UK) presenting with RFM after 36 weeks gestation between October 2011 and August 2012 were invited to participate. The protocol was given a favourable ethical opinion by the Greater Manchester North Research Ethics Committee (11/NW/0664) and was registered on the International Standard Randomised Controlled Trial Number Register (ISRCTN07944306). Women were excluded if there was a known congenital anomaly, multiple pregnancy, the fetus required immediate delivery for abnormal fetal heart rate trace, maternal age was less than 16 years or they were unable to give informed consent.

### Recruitment

Women meeting the inclusion criteria were approached by the research midwife (GB) either during their attendance at the maternity day unit (MDU) if between 9 am–5 pm or on the morning after attending the obstetric triage service outside normal hours (5 pm–9 am). Women approached during attendance at MDU were given information during the initial fetal heart rate assessment which lasted approximately 45 minutes. Women were then randomised to standard or intensive management groups by computer, using individual randomisation in a 1:1 ratio with random variable block size. This was facilitated by the University of Nottingham Clinical Trials Unit. We aimed to randomise 120 patients in total, giving 60 patients in each arm of the study. From our previous studies we anticipated a loss to follow-up of <5% (3 participants per arm) and estimated that 150 women would need to be approached to recruit 120 participants. In addition, demographic data were collected on non-participants to determine whether there were differences between participants and non-participants.

### Intervention

Women assigned to intensive management had an ultrasound scan to measure head circumference, abdominal circumference and femur length, liquor volume and umbilical artery Doppler. Estimated fetal weight (EFW) was calculated using the Hadlock B formula [14]. hPL was measured in maternal serum by enzyme-linked immunoassay of samples in triplicate (Immunodiagnostic systems, Boldon, UK). Low hPL for gestation was defined as <0.8 MoM. If any of the investigations were abnormal, obstetricians caring for women in the intensive group were recommended to expedite delivery by the most appropriate method, usually induction of labour unless there was an indication for Caesarean delivery. Women in the standard group had assessment of EFW, liquor volume and umbilical artery Doppler if they met the unit criteria for ultrasound scan which were: two or more attendances with RFM or more than 37 completed weeks’ gestation or symphysiofundal height below the tenth centile. The results of these investigations were discussed with the obstetrician caring for the woman who made an independent decision regarding subsequent management.

### Participant outcome

All outcome data for trial analysis were obtained from the clinical notes. Data were collected on trial-specific case report forms according to a standard operating procedure and entered into a Microsoft Access database by the research midwife. Ten percent of trial records selected at random were checked for accuracy by a second investigator (AH). Poor pregnancy outcome was defined as stillbirth, small for gestational age infant defined as an individualised birthweight centile <10^th^, umbilical arterial pH <7.1 or unexpected admission to NICU. These outcomes were chosen as previous studies have shown that infants stillborn after RFM were SGA [15], and infants subject to severe intra-uterine compromise might not die but instead require neonatal intensive care [16]. Data regarding levels of maternal anxiety were obtained before and after the investigations by the state-trait anxiety index (STAI) which has previously been validated in pregnancy. Specifically-designed questionnaires were designed to assess women’s and clinician’s experiences of trial procedures. The women’s questionnaire contained scales to rank responses to statements about participation in the trial, two closed questions and a free-text box for further comments. The questionnaire was piloted with five women and responses checked for clarity and content validity. The questionnaire for clinicians assessed their awareness of the trial with closed questions and their opinions and experiences of the trial with scales to rank their responses. The questionnaires were administered after women had given birth and in the case of clinicians after the feasibility study had been completed. STAI and questionnaire responses were collated in Microsoft Excel.

### Analysis

Participant demographics, adherence to protocol and participant questionnaires were analysed descriptively in accordance with published recommendations for analysis of pilot studies [17]. State anxiety scores before and after investigations were compared using Wilcoxon signed rank test; trait anxiety scores and changes in state scores were compared between groups using Mann-Whitney U tests. All analyses were conducted using SPSS version 16.0 (SPSS Inc, IL, USA), a p-value of <0.05 was considered to be statistically significant in all analyses.

## Results

### Participant recruitment and retention

**Figure 1:**
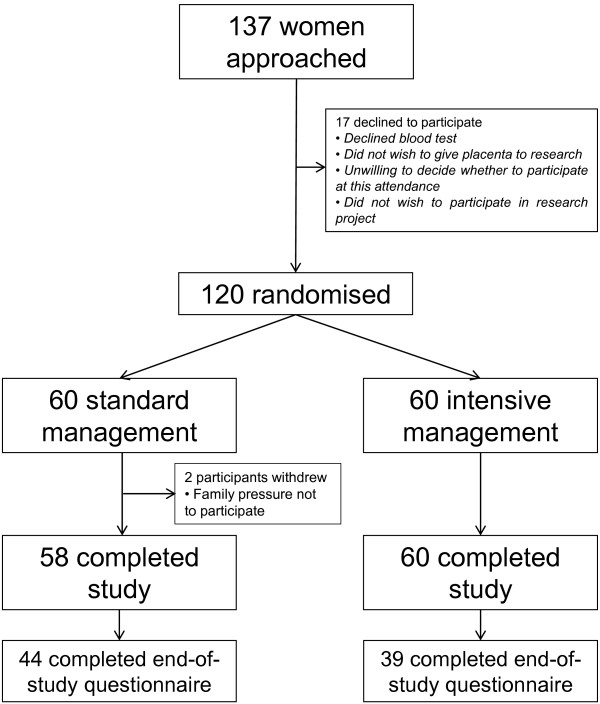
Flow diagram of the trial process.

One hundred and thirty seven women were approached to participate in the study between December 2011 and August 2012 (Figure 1). One hundred and twenty (87.5%) agreed and 60 were randomised to each arm. Women who chose not to participate in the study had similar demographic characteristics to participants except for a higher proportion of women of Black African ethnicity in non-participants (Table 1). However, women participating in this study had similar demographic characteristics to the whole unit population. This was also the case in a previous cohort study carried out in the same institution [10]. Two patients (1.7%) withdrew from the study, both of whom were in the standard treatment arm; these women cited pressure from partners not to participate as motivating their decision to withdraw from the study.

**Table 1:** Demographic characteristics of study participants compared to those would did not consent and participants in a previous cohort study in the same centre

**Characteristic**	**Participants (n = 120)**	**Non-consenters (n = 17)**	**All births at unit during study period (n = 7,533)**
Age	28 (18–42)	27 (19–37)	29 (13–46)
BMI	25 (18–50)	26 (15–38)	25 (14–63)
Gravidity	2 (1–9)	2(1–4)	-
Parity	1 (0–5)	0 (0–2)	0 (0–15)
Ethnicity			
*Bangladeshi*	*6 (5%)*	*1 (6%)*	134 (2%)*
*Black African*	*8 (7%)*	*7 (41%)*	612 (9%)*
*Black Caribbean*	*3 (3%)*	*1 (6%)*	138 (2%)*
*Indian*	*4 (3%)*	*3 (18%)*	191 (3%)*
*Pakistani*	*19 (16%)*	*1 (6%)*	865 (13%)*
*White European*	*74 (62%)*	*2 (12%)*	4,875 (71%)*
*Other ethnic groups*	*6 (5%)*	*2(12%)*	50 (1%)*
Cigarette Smoker	12 (10%)	Unknown	723 (10%)^†^
Gestation at Presentation	38^+6^ (36^+0^–41^+1^)	38^+4^ (36^+2^–40^+5^)	-

For continuous variables the median is shown with the range in parentheses, for categorical variables the number of cases is shown with percentage in parentheses. *Denominator for ethnicity in unit population = 6,865. ^†^Denominator for smoking status in unit population = 7,512.

### Participant acceptability

**Figure 2:**
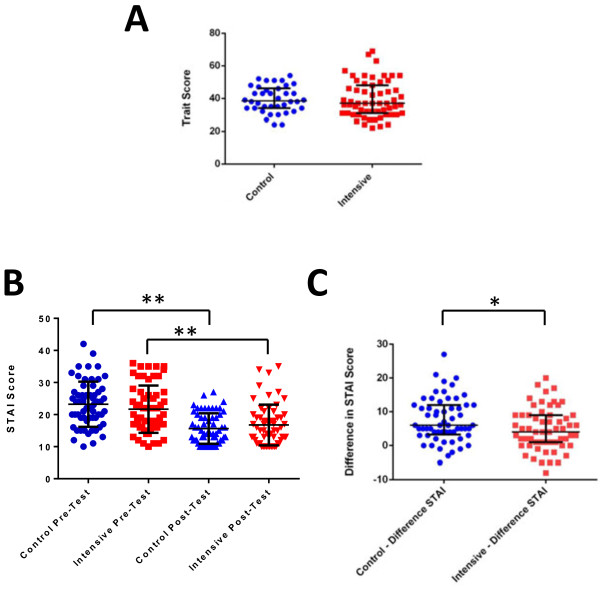
**State-trait anxiety scores for participants (n = 120). A**) There was no difference in the trait score between participants randomised to the control or intensive management protocol. **B**) Maternal state anxiety was significantly reduced in both control and intensive management groups after completion of the investigations (** p < 0.01). **C**) The reduction in state anxiety was greatest in the women in the control group (* p < 0.05).

There was no difference in the anxiety score before investigations between women assigned to the control or intensive management groups (Figure 2A). Women in both groups experienced a reduction in state anxiety after investigations (Figure 2B); this reduction in state anxiety was greater in women in the control group (Figure 2C). This may either be a chance finding or could be attributed to the increased detection of abnormalities requiring intervention in the intensive management group. Eighty-three participants (69%) responded to the end of study questionnaire given after the birth of their child; 44 (53%) of respondents received standard care and 39 (47%) of respondents received intensive management. All participants felt that the time at which they were approached to participate was acceptable (Table 2); 99% and 98% of participants felt they had sufficient information and time to make a decision to participate respectively. All patients understood the patient information and consent form. The majority of participants did not mind which group they were assigned to, although 6 (7%) expressed a clear preference to enter the intervention arm. Eighty-one (98%) participants felt that the number of tests to establish fetal wellbeing was satisfactory; the remaining two participants would have liked more tests, these participants were both in the standard management group. Seventy-seven (93%) of respondents felt the results were given in a timely manner. Overall, 98% of participants would recommend the study to a friend whose baby was moving less.

**Table 2:** Responses from participants to the end of study questionnaire administered after delivery (n = 83)

**Statement/Question**	**Strongly agree**	**Agree**	**Unsure**	**Disagree**	**Strongly disagree**	**Not answered**
The time I was first approached to participate in the trial was acceptable	52 (63)	31 (37)	0 (0)	0 (0)	0 (0)	0 (0)
I had sufficient information to make a decision to participate in the trial	56 (67)	26 (31)	1 (1)	0 (0)	0 (0)	0 (0)
I had sufficient time to make a decision to participate in the trial	46 (55)	33 (40)	1 (1)	1 (1)	0 (0)	2 (2)
I understood the information I was given	52 (63)	31 (37)	0 (0)	0 (0)	0 (0)	0 (0)
The consent form was clear	53 (64)	30 (36)	0 (0)	0 (0)	0 (0)	0 (0)
I did not mind which group (standard management or intensive testing) I was assigned to	36 (43)	34 (41)	6 (7)	6 (7)	0 (0)	1 (1)
The amount of tests I had was satisfactory	45 (54)	34 (41)	0 (0)	2 (2)	0 (0)	2 (2)
The results of the tests were given within an appropriate time period	50 (60)	27 (33)	4 (5)	0 (0)	0 (0)	2 (2)
	**Yes**		**Unsure**	**No**		**Not answered**
Would you recommend participating in the trial to a friend who noticed her baby was moving less	81 (97.6)		2 (2.4)	0 (0)		0 (0)

Figures in parentheses denote percentages of respondents.

### Protocol adherence and professionals’ views

All patients in the intensive management arm had an ultrasound assessment of fetal growth, liquor volume and umbilical artery Doppler as compared to 97% in the control group. All women in the intensive group had hPL measured in maternal serum compared to no patients in the control group (Table 3). Ultrasound scans showed reduced fetal growth, oligohydramnios or abnormal umbilical artery Doppler in 14% of cases, 20% of the intensive group and 8% of the control group; as randomisation occurred before the ultrasound scan was performed this discrepancy must be due to chance. hPL was <0.8 MoM in 30% of women in the intensive management group. Overall, the frequency of induction of labour (IOL) was no different between groups (Table 3); 59% in control compared to 62% in intensive management group. However, the proportion of women being induced for RFM was higher in the intensive management group 50% vs. 26%. In the control group the majority of IOL were for prolonged rupture of membranes or prolonged pregnancy. There was no difference in gestational age at presentation or at delivery. There was no increase in instrumental or Caesarean birth rates. Perinatal outcomes are shown in Table 4. Overall, 20% of women had a poor perinatal outcome, with the most frequent outcome being birthweight <10^th^ centile; there were no stillbirths in either group. Although the study was not powered to detect differences in outcomes, and no formal hypothesis tests were planned; there were fewer poor perinatal outcomes in the intensive management group. It is interesting that the increased number of abnormalities on the ultrasound scan observed in the intensive group did not translate into increased poor pregnancy outcomes.

**Table 3:** Protocol compliance as assessed by proportion of women having investigations and induction of labour (IOL)

**Intervention**	**Control (n = 60)**	**Intensive (n = 60)**	**Total (n = 120)**
Ultrasound Scan	58 (97%)	60 (100%)	118 (98%)
Abnormal EFW, LV or UAD	5 (8%)	12 (20%)	17 (14%)
hPL	0 (0%)	60 (100%)	60 (50%)
Abnormal hPL (<0.8 MoM)	-	18 (30%)	18 (15%)
	**Control (n = 58)**	**Intensive (n = 60)**	**Total (n = 118)**
IOL	34 (59%)^†^	37 (62%)	71 (60%)
Indication for IOL			
RFM	15 (26%)^†^	30 (50%)^‡^	45 (38%)
Cholestasis	1 (2%)	1 (2%)	2 (2%)
Hypertension/Preeclampsia	0	1 (2%)	1 (1%)
Polyhydramnios	1 (2%)	0	1 (1%)
Prolonged pregnancy	4 (7%)	2 (3%)	6 (5%)
Prolonged rupture of membranes	10 (17%)	3 (5%)	13 (11%)
Small for gestational age	2 (3%)	0	2 (2%)
Suspicious cardiotocograph on subsequent presentation	1 (2%)	0	1 (1%)
Gestation at Delivery	40^+1^ (37^+0^–42^+1^)	39^+6^ (36^+4^–42^+0^)	40^+0^ (36^+4^–42^+1^)
Delivery by CS	8 (14%)^†^	4 (7%)	12 (10%)
Instrumental Delivery	11 (19%)^†^	15 (25%)	26 (22%)

Absolute values are shown with percentages in parentheses. † = Denominator value of 58 (as two outcomes unknown). ‡ The 30 women induced for RFM in the intensive arm all had either abnormal scan findings or low hPL.

**Table 4:** Perinatal outcome in participants in control and intensive groups

**Intervention**	**Control (n = 58)**	**Intensive (n = 60)**	**Total (n = 120)**
Poor Pregnancy Outcome	17 (29%)^†*^	7 (12%)*	24 (20%)
*Primary*	*6 (10%)*^*†*^	*2 (3%)*	*8 (7%)*
*Stillbirth*	*0 (0)*	*0 (0)*	*0 (0)*
*Unexpected Admission to NICU*	*3 (5%)*^*†*^	*1 (2%)*	*4 (3%)*
*Metabolic acidosis (art pH ≤7.1)*	*3 (5%)*^*†*^	*1 (2%)*	*4 (3%)*
*Secondary*			
*Birthweight ≤ 10*^*th*^*centile*	*11 (19%)*^*†*^	*5 (8%)*	*16 (14%)*

* Intensive vs. Control p = 0.022 (Fisher’s Exact test).

Eight consultant obstetricians and seven experienced midwives from the maternity day unit, maternity triage and delivery unit responded to the staff questionnaire (Table 5). Fourteen staff (93%) were confident in which investigations to use and 87% confident when to expedite delivery when caring for women with RFM. However, only 33% felt that care of women with RFM is currently based on grade 1/2 evidence. All staff were aware that the study had taken place, 87% were aware that women in their care had participated in the study and 73% were aware of the recommended management plan. Ten (67%) of professionals felt that results were given in a timely manner, 60% felt that the results of the investigations for patients in the intensive group altered their management with 27% perceiving an increase in labour interventions. Twenty percent of professionals felt that the study increased workload to the antenatal service, but the majority (47%) disagreed with this statement. All professionals would offer participation in this trial to a woman presenting with RFM.

**Table 5:** Responses from obstetricians and midwives to the questionnaire administered after completion of the study (n = 15)

**Statement/Question**	**Strongly agree**	**Agree**	**Unsure**	**Disagree**	**Strongly disagree**
I am confident which investigations to use when caring for women with RFM	6 (40)	8 (53)	0 (0)	1 (7)	0 (0)
I am confident when to intervene (expedite delivery) when caring for women with RFM at term	6 (40)	7 (47)	1 (7)	1 (7)	0 (0)
The care of women (clinical and supportive management) with RFM is currently based on robust evidence (Grade 1 or 2).	2 (13)	3 (20)	2 (13)	6 (40)	2 (13)
The results of the investigations were given to women and the medical team in an appropriate time period	3 (20)	7 (47)	5 (33)	0 (0)	0 (0)
The findings of the investigations (ultrasound scan and human placental lactogen) in the ReMIT study altered my management of women with RFM	3 (20)	6 (40)	6 (40)	0 (0)	0 (0)
I appeared that participation in the ReMIT study increased anxiety in the participants	0 (0)	1 (7)	3 (20)	9 (60)	2 (13)
In my opinion the ReMIT study increased labour interventions (e.g. induction of labour, Caesarean section)	1 (7)	3 (20)	8 (53)	2 (13)	1 (7)
The ReMIT study led to a significant increase in the workload to the antenatal triage, maternity day unit and delivery suite	2 (13)	1 (7)	5 (33)	6 (40)	1 (7)
	**Yes**		**Unsure**	**No**	
Were you aware that the ReMIT study was taking place in St Mary’s Hospital?	15 (100)		0 (0)	0 (0)	
Were any of the women in your care participants in the ReMIT study?	13 (86.7)		0 (0)	2 (13.3)	
Were you aware of the recommended management plan for the women in the intensive arm of the ReMIT study?	11 (73.3)		0 (0)	4 (26.7)	
Would you be happy to offer participation in this trial to a woman who noticed her baby was moving less	15 (100)		0 (0)	0 (0)	

Figures in parentheses denote percentages of respondents.

## Discussion

A recent systematic review and meta-analysis and the RCOG guideline have identified the need for robust evidence to guide the management of RFM [5,7]. This feasibility study suggests that a randomised controlled trial of the individualised management of RFM would be acceptable to women perceiving RFM after 36 weeks gestation and to professionals, given a participation rate of 87% and a dropout rate of <2%. A minority of participants were keen to be randomised to the more intensive management, but this did not appear to cause participants to withdraw from the standard arm of the trial. These effects of participant preference are similar to those previously described in other analyses of randomised controlled trials [18,19]. Importantly, both strategies tested here resulted in a decrease in maternal anxiety after tests were performed, but the reduction in anxiety was greater in women receiving standard management compared to intensive management group. This may reflect concerns of women who had an abnormal investigation leading to IOL which was more frequent in the intensive management group. This feasibility study suggests that a larger trial may increase the rate of IOL for RFM as a primary intervention for abnormal investigation findings, but this should not impact on instrumental or Caesarean delivery. This is in agreement with studies that describe no increased rate of obstetric intervention in RCT of fetal movement counting or in quality improvement studies for women with RFM [20,21].

Clinicians in this single tertiary centre were confident in their current management of RFM, but 66% recognised that their management strategies were not based on robust evidence. Studies of the management of RFM in UK and Australia highlighted variation in individuals’ management of RFM and that a large proportion of respondents would be willing to participate in a trial of fetal movement counting or management of RFM [8,9]. However, almost all of the participants in this clinical trial underwent ultrasound to assess fetal growth and liquor volume irrespective of the group they were assigned to. We suspect that this reflects the local practice within our tertiary unit, where there is an interest in using RFM to identify compromised fetuses, as surveys in the UK and Australia found that 18.6%–20.2% of obstetricians routinely carried out ultrasound assessment of fetal growth. A recent survey of guidelines for the management of RFM in obstetric units (Whitworth et al. unpublished data) has found that a minority of local guidelines (33%) recommend ultrasound scan as a routine investigation for RFM. Prior to commencing a larger multi-centre study, clinical practice should be re-evaluated to determine standard management of RFM and the equipoise of practitioners regarding the investigations and management of women presenting with RFM.

This feasibility study found that some patients, particularly those of Black African ethnicity were more likely to decline participation. It is difficult to draw firm conclusions due to small numbers in this study, but this needs to be investigated further to determine whether members of minority ethnic groups would be less likely to participate in a trial of the management of RFM and if so, the reasons underlying their decision. Although participants felt that the trial documentation was clear, this may need to be adapted to ensure that entry to the trial is as inclusive as possible. Furthermore, participants could be reassured there was no increased obstetric interventions in participants in the intensive arm of the trial and that few patients discontinued the trial.

This study confirmed evidence from prospective and retrospective cohort studies [10,15] that approximately 20% of women presenting with RFM will have a poor pregnancy outcome, defined here as a composite of: birthweight <10^th^ centile on a customised birthweight chart, unexpected admission to NICU or umbilical arterial pH <7.1. There were no stillbirths or neonatal deaths in this small RCT as would be expected given the sample size (the background incidence of stillbirth in this institution is 6.9/1,000 live births). This observation raises two related questions with regard to the sample size of a definitive trial: i) the use of a composite outcome and ii) the subsequent formulation of the question to be addressed.

Due to the relative infrequency of stillbirth, currently 5.2/1,000 live births in the UK, studies frequently use a composite outcome. For example the composite outcome used in the DIGITAT study, a randomised controlled trial comparing induction of labour to expectant management for intrauterine growth restriction near term, was: death before hospital discharge, five minute Apgar score of less than 7, umbilical artery pH of less than 7.05, or admission to neonatal intensive care [22]. However, other studies of groups at high-risk of perinatal mortality, such as severe FGR have used death before 2 years of age e.g. the GRIT study [23]. The use of composite end points in RCTs is controversial; they are used to reduce sample size requirements and capture the overall impact of an intervention [24], but they may exaggerate the effect of an intervention, particularly where one component of the composite outcome, which may be less biologically relevant, shows a significant effect [25]. It is essential that a composite outcome contains outcomes important to patients and the magnitude of effect is maintained across different individual endpoints within the composite outcome [26]. Our preliminary data suggest that the composite “poor perinatal outcome” used in this study: stillbirth, small for gestational age infant defined as an individualised birthweight centile <10^th^, umbilical arterial pH <7.1 or unexpected admission to the neonatal intensive care unit, encompasses the spectrum of morbidity and mortality associated with RFM, which may result from the relationship between RFM and placental insufficiency. The most frequent endpoint is birthweight <10^th^ centile which was a secondary outcome in this feasibility study. However, in this study 7% of patients had a poor primary outcome including acidosis or admission to NICU. Prior to a definitive trial further work on composite outcomes is required to address whether the effect is maintained across different individual endpoints within the composite outcome.

The formulation of the question to be addressed is also critical to determine sample size. Alternatives to individual management include a cluster-randomsied or stepped-wedge design. Here the choice to participate is removed from the individual, but this design may address relevant confounding effects in the management of RFM e.g. a high proportion of the standard group had an ultrasound scan. However, one drawback of such studies is the need for greater numbers of participants. An alternative strategy proposed to reduce the sample size required for a definitive RCT of the management of RFM is to alter the question being posed and randomise to intervention after having a screening test [27]. In this context, women determined to be high-risk for poor perinatal outcome after RFM would be randomised to intervention (delivery) or conservative management. While this study has demonstrated that women presenting with RFM and the clinicians caring for them will accept an RCT randomising them to intensive or standard investigations, a further feasibility study would be necessary to determine whether randomisation after a test designating participants to be at high-risk of complications would be feasible. As women in the intensive arm already experienced greater anxiety than women receiving standard care, it could be speculated that delaying intervention after highlighting an increased risk of morbidity would raise maternal anxiety to unacceptable levels.

To determine sample size of a definitive trial the following assumptions have been made: i) 33% of stillbirths occur after 36 weeks gestation [28]; ii) the risk of stillbirth after RFM is three-times greater than the background risk; iii) 20% of pregnancies with RFM end in poor pregnancy outcome. To detect a 20% reduction in stillbirths after 36 weeks gestation with a power of 80% and α = 0.05 would require 146,945 patients per arm, if this is extended to death before discharge, 128,457 patients per arm are required. To use the approach of randomisation after testing conservatively assumes that women with a positive test are at a four-fold increased risk of poor perinatal outcome. To demonstrate a 20% reduction in perinatal mortality would require 5,568 participants in each arm, as 50% of women test positive, 22,272 women would need to be approached and tested. In comparison, to observe a reduction in composite poor perinatal outcome from 20% of women presenting with RFM after 36 weeks gestation to 16% would require 1,447 participants in each arm. Using the randomisation after testing approach in combination with the measure of poor perinatal outcome would reduce this further to 270 participants per arm, meaning that 1,080 participants would be required. There are approximately 700,000 births in England and Wales per year [29], and assuming 6% of women are reported to attend on at least one occasion with RFM [1], there are estimated to be 42,000 women presenting with RFM in the third trimester of pregnancy. If 33% of these are over 36 weeks gestation, this leaves approximately 14,000 women who may be eligible for a multi-centre study. This makes a study with stillbirth as the sole primary outcome unfeasible. However, using a composite primary outcome or randomisation after testing approach appropriate recruitment would be achievable. The feasibility study carried out here suggests that the former would be acceptable to patients and professionals. However, additional measures may be required to reduce maternal anxiety due to abnormal investigations the intensive group; these could include verbal and written information provided by staff.

## Conclusion

This feasibility study demonstrates that an individual randomised controlled trial of the management of RFM is feasible in a diverse group women perceiving RFM after 36 weeks gestation. Investigations decrease maternal anxiety. Participants in the intensive group were more likely to have IOL for RFM although the overall rates were similar. Professionals described the need for better evidence on which to base practice and were willing to adapt their management strategies to the trial protocol. Further studies are required to determine the likely level of intervention in the standard care arm in multiple centres, to develop additional placental biomarkers and to confirm that the composite outcome is valid.

## Abbreviations

EFW: Estimated fetal weight; FGR: Fetal growth restriction; hPL: Human placental lactogen; IOL: Induction of labour; NICU: Neonatal intensive care unit; RCOG: Royal College of Obstetricians and Gynaecologists; RCT: Randomised controlled trial; RFM: Reduced fetal movements; SGA: Small for gestational age; STAI: State trait anxiety index.

## Competing interests

The authors declare that they have no competing interests.

## Authors’ contributions

AEPH, SAR, MKW, EDJ and TL designed and secured funding for the study. AH obtained ethical approval for the study. GB approached and recruited participants, performed ultrasound scan and obtained blood for analysis. AG analysed hPL in maternal serum. GB and AH obtained outcome data from maternal case notes. AH and SR performed data analysis. JG chaired the trial steering group. All authors contributed to the writing and review of the manuscript. All authors have read and approved the final manuscript.

## Pre-publication history

The pre-publication history for this paper can be accessed here:

http://www.biomedcentral.com/1471-2393/13/95/prepub
